# Chemical Composition, Antibacterial and Antifungal Activities of Crude *Dittrichia viscosa* (L.) Greuter Leaf Extracts

**DOI:** 10.3390/molecules22070942

**Published:** 2017-06-30

**Authors:** Wafa Rhimi, Issam Ben Salem, Davide Immediato, Mouldi Saidi, Abdennacer Boulila, Claudia Cafarchia

**Affiliations:** 1 Faculté des Sciences de Bizerte, Université de Carthage, 7021 Zarzouna, Tunisia; wafa_Rhimi@hotmail.com; 2 Laboratory of Biotechnology and Nuclear Technolog, National Centre of Nuclear Science and Technology (CNSTN), Sidi Thabet Technopark, 2020 Ariana, Tunisia; official@cnstn.rnrt.tn; 3 Dipartimento di Medicina Veterinaria, Università degli Studi di Bari, 70010 Valenzano, Italy; direzione.veterinaria@uniba.it (D.V.); claudia.cafarchia@uniba.it (C.C.); 4 Laboratory of Natural Substances LR10INRAP02, National Institute of Research and Physico-chemical Analyses, Biotechpole of Sidi Thabet, 2020 Ariana, Tunisia; abdennacer.boulila@inrap.rnrt.tn

**Keywords:** *Dittrichia viscosa*, antifungal activities, *Candida* spp., *Malassezia* spp., *Microsporum canis*, *Aspergillus fumigates*

## Abstract

The small amount of data regarding the antifungal activity of *Dittrichia viscosa* (L.) Greuter against dermatophytes, *Malassezia* spp. and *Aspergillus* spp., associated with the few comparative studies on the antimicrobial activity of methanolic, ethanolic, and butanolic extracts underpins the study herein presented. The total condensed tannin (TCT), phenol (TPC), flavonoid (TFC), and caffeoylquinic acid (CQC) content of methanol, butanol, and ethanol (80% and 100%) extracts of *D. viscosa* were assessed and their bactericidal and fungicidal activities were evaluated. The antibacterial, anti-*Candida* and anti-*Malassezia* activities were evaluated by using the disk diffusion method, whereas the anti-*Microsporum canis* and anti-*Aspergillus fumigatus* activities were assessed by studying the toxicity effect of the extracts on vegetative growth, sporulation and germination. The methanolic extract contained the highest TPC and CQC content. It contains several phytochemicals mainly caffeoylquinic acid derivatives as determined by liquid chromatography with photodiode array and electrospray ionisation mass spectrometric detection (LC/PDA/ESI-MS) analysis. All extracts showed an excellent inhibitory effect against bacteria and *Candida* spp., whereas methanolic extract exhibited the highest antifungal activities against *Malassezia* spp.*, M. canis* and *A. fumigatus* strains. The results clearly showed that all extracts, in particular the methanolic extract, might be excellent antimicrobial drugs for treating infections that are life threatening (i.e., *Malassezia*) or infections that require mandatory treatments (i.e., *M. canis* or *A. fumigatus*).

## 1. Introduction

The growing worldwide concern about the alarming increase in the rate of human and animal infections caused by antibiotic-resistant microorganisms have spurred the interest of the scientific community in developing alternative methods for their control [1]. Many kinds of natural extracts from medicinal plants containing phenolic and flavonoid compounds have excellent biological properties and are used as alternative therapies. Among the large variety of Mediterranean folkloric herbs, *Dittrichia viscosa* belonging to the Asteraceae family, has proven to be a source of natural products forming the basis for alternative medicine and natural therapies [2,3,4]. *Dittrichia viscosa* was studied against antibiotic-resistant microorganisms, antibacterial activity and anti-fungal activity against *Candida albicans* and *Fusarium* species [5,6]. To the best of our knowledge, reports on antifungal activity of *D. viscosa* against dermatophytes, *Malassezia* spp. and *Aspergillus* spp. are scant or limited to *Microsporum canis*. In particular, dermatophytes are a group of fungi which have the ability to invade the keratinized tissues (skin, hair, nails) causing cutaneous infections in humans and animals commonly known as dermatophytosis [7]. They are distributed worldwide and some of them are considered zoonotic, being transmitted from animals to humans [8]. The treatment of infections is mandatory due to the contagious and the zoonotic nature and usually requires long antifungal therapy with azoles [9]. In addition, these treatments are not usually performed in food producing animals since they are more expensive, and treated animals need long withholding before using in food processing industry [10].

The fungal genus *Malassezi*a is part of the normal skin microbiota. These yeasts cause human and animal skin disorders in immune-competent hosts and systemic infections in immune- compromised patients which usually require prolonged treatment with and/or high doses of antifungal agents [11,12]. In addition, recent studies clearly show that the same species within the genus of *Malassezia furfur* and *Malassezia pachydermatis* are characterized by high minimal inhibitory concentration (MIC) values against all azole drugs commonly employed in the treatment of the infections.

Finally, *Aspergillus* species are found worldwide in humans and in almost all domestic animals and birds as well as in many wild species, causing a wide range of diseases from localized infections to fatal disseminated diseases, as well as allergic responses to inhaled conidia [13]. Some prevalent forms of animal *aspergillosis* are invasive fatal infections and are difficult to treat. In addition, the environmental diffusion of *A. fumigatus* strains presenting azole resistant phenomena is worldwide reported [13].

Thus, this study aimed to: (i) quantify the phenolic and flavonoids content of *D. viscosa* leaf extract with different solvents; (ii) evaluate their activities against gram positive and negative bacteria, and against *Candida* spp. (i.e., *Candida albicans*, *Candida krusei*, *Candida prapsilos*); and (iii) to assess their activities against *Malassezia* spp. (*Malassezia pachydermatis* and *Malassezia furfur*), *Aspergillus fumigatus* and *Microsporum canis*.

## 2. Results and Discussion

### 2.1. Phytochemical Screening

The total condensed tannin (CTC), phenol (TPC), flavonoid (TFC), and caffeoylquinic acid (CQC) content of different *D. viscosa* extracts are reported in Table 1. They are expressed as mg catechin equivalent (CE), mg gallic acid equivalent, mg quercetin equivalent (QE) and mg of chlorogenic acid equivalent (ChlA E) per g dry extract, respectively.

The CTC amounts varied from 7.05 ± 1.6 to 27.15 ± 2.21 mg CE/g, being the highest in the methanolic extract (Table 1). The TPC ranged from 75.34 ± 1.30 to 123.39 ± 1.22 mg GAE/g, the highest content retrieved in methanolic and 80% ethanolic extracts (Table 1).

The CQC amounts of *D. viscosa* extracts ranged from 57.11 ± 0.98 to 87.61 ± 1.06 mg ChlA E/g (Table 1) and the highest amount of CQC was registered in methanolic extract. The TFC varied from 30.86 ± 1.28 to 58.03 ± 1.85 mg QE/g and the highest content was registered in butanolic extract (Table 1). The methanolic extract contains the highest CTC, TPC and CQC values while the butanolic extract contained the highest amount of TFC.

The results of this study clearly indicate that phenolic and flavonoids content of *D. viscosa* crude extracts vary according to the solvent extraction procedure. In particular, this study reports for the first time the presence of condensed tannins in this plant species. Indeed, no previous studies have evaluated CTC in *D. viscosa* leaves, but results herein indicate that the amount within methanolic extracts are in the same range as those of some *Asteraceae* species such as *Artemisia* genus [14]. On the contrary, the TPC values of Tunisian *D. viscosa* extracts were in same range or slightly lower than those reported from Turkish or Moroccan samples [4,15], thus suggesting that TPC values of *D. viscosa* does not vary accordingly to plant origin. Accordingly, the TPC amounts depend on the polarity of the solvent, and it was highest when the solvent polarity increased. Similar results were reported by Negi and Jayaprakasha when studying methanol extracts of *Punica granatum* peel [16]. However, a high value of TFC in our extracts was detected in butanolic extract, suggesting that the flavonoid composition of *D. viscosa* might comprise of substances with a high solubility in butanol, like luteolin derivatives [17,18].

### 2.2. Phenolic Profile of D. viscosa Extracts

**Figure 1:**
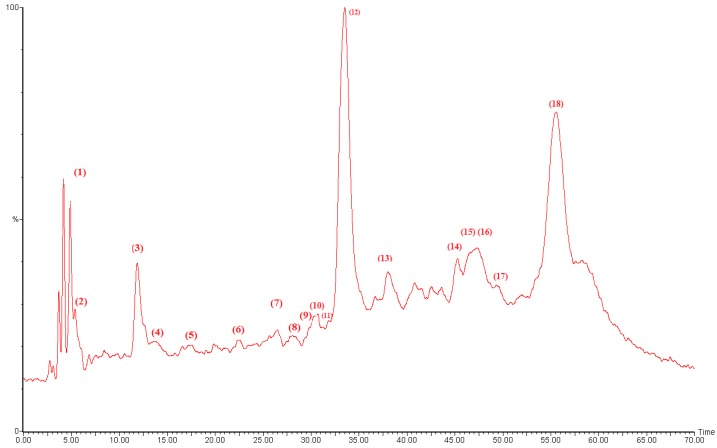
Chemical characterization of methanolic extract of *D. viscosa* leaves by HPLC-PDA-ESI-S. The peaks are numbered and assignments are given in Table 2.

The HPLC-PDA/ESI-MS analysis allowed us to tentatively identify 18 phenolic compounds in the methanolic *D. viscosa* extract (Figure 1). The phenolic fraction of methanolic *D. viscosa* extract was dominated by caffeoylquinic acid derivatives such as chlorogenic acid, dicaffeoylquinic acid isomers, and caffeoyl glucose as it shown in Table 2. Other hyhydroxycinnamic acids like coumaric acid and caffeic acid derivatives were also detected.

Some flavonoid compounds were also detected. They were represented exclusively by quercetin derivatives (e.g., quercetin-*O*-hexoside, quercetin glucuronide, quercetin dimethyl ether isomers), and the flavonol catechin glucoside. The identification of phenolic compounds by HPLC-PDA-ESI-MS as shown in Table 2 confirmed our photochemical screening findings about the richness of *D. viscosa* extract in caffeoylquinic acid derivatives, and in agreement with previous reports from Israel, Turkey and Tunisia [4,15,19]. From these results, it emerges that *D. viscosa* might be advised as potential source of bioactive components especially caffeoylquinic acid derivatives.

### 2.3. Antibacterial, Anti-Candida and Antifungal Activity of D. viscosa Extracts

Table 3 shows the inhibitory effects of *D. viscosa* extracts against Gram positive (i.e., *Staphylococcus aureus*, *Enterococcus feacium*, *Streptococcus agalactiae*) and Gram negative bacteria (i.e., *Escherichia coli* and *Salmonella typhimurium*) with the inhibition halo ranging from 9.5 to 34.5 mm. No statistically significant differences were recorded between different extracts. The highest antimicrobial activity was observed against *Enterococcus feacium* (G+) and *Streptococcus agalactiae* (G+) with inhibition zones of 34.5 ± 0.7 mm and 29 ± 1.41 mm, respectively.

The results of anti-*Candida* and anti-*Malassezia* activities are reported in Table 4. The diameter halo ranged from 7 to 14.5 mm according to extract concentration. No significant differences were recorded among the activity of different extracts against *Candida* species.

Regarding the biological activity, the results herein are not only confirmed existing data about the antibacterial activities of crude extracts of *D. viscosa,* but are extended our knowledge on the antifungal activities against different *Candida* spp. (i.e., *C. parapsilosis* and *C. krusei*), *Malassezia* and *A. fumigatus* strains.

All the extracts investigated exhibited antibacterial and anti-*Candida* activities which are independent of the extraction solvent, but dependent on the extract concentrations, suggesting that both flavonoid and phenolic compounds might act as antibacterial and anti-*Candida* drugs [20]. It is well known, that luteolin derivatives, isorhamnetin and in particular 3′-di-*O*-methylquercetin and 3-*O*-methyquercetin from Jordanian *D. viscosa* have an excellent inhibitory efects against *B. cereus*, *S. typhimurium* and *S. aureus.* Phenolic compounds such as hydroxycinnamic acids derivatives (caffeoylquinic acid and chlorogenic acid) or *p-*coumaric acid are also potent inhibitors of *E. coli*, *K. pneumoniae*, *B. cereus* and *C. albicans* [20,21]. Both phenolic and flavonoid compounds provoke damage in bacterial or yeast cell walls and cytoplasmic membranes [21,22]. Interestingly, the gram-positive bacteria tested were significantly more sensitive to *D. viscosa* extracts than gram-negative bacteria, most likely due to the presence of a lipopolysaccharide (LPS) membrane in Gram-negative bacteria, being more resistant to the foreign agents [23]. The absence of these LPS in membrane cell of *Candida* spp. makes them vulnerable against foreign agents.

The anti-*Malassezia* inhibition zone ranged from 0 to 11 mm. Among the yeast populations tested in this study, *Malassezia* species present a susceptibility profile varying according to the species and strain (Table 4). In particular, all extracts showed good broad-spectrum action against *M. pachydermatis* from dog otitis/dermatitis whereas the lowest effectiveness against *Malassezia furfur* isolated from human blood stream infections. These results are not surprising since similar trends were observed when the susceptibility of *M. pachydermatis* and *M. furfur* to azoles was compared due to the variability of the cell wall chemical composition of *Malassezi*a yeasts [24]. The anti-*Malassezia* activity of our extracts not only varied according to *Malassezia* species, but also to the solvent used for extraction with methanol extract most active against *M. furfur* (Table 4). Indeed, the extracts prepared with the high polarity solvents (methanol) were more effective against *Malassezia* species including *M. furfur* than those using low polarity solvents. Similar trends have been observed using chloroformic extract of *Lawsonia inermis* leaves or aqueous extracts of *Allium cepa* and *Allium sativum* against *Malassezia furfur* [25]. The anti-*Malassezia* activities of *D. viscosa* extracts may be explained by the high TFC and CQC content identified in methanol extracts thus confirming previous results with *I. paraguariensis* extracts [26].

Toxicity assays and the effect on fungal germination of extracts against *M. canis* and *A. fumigatus* are reported in Table 5 and Table 6, respectively. The germination and sporulation were expressed as mean values (±standard deviation) of Log10 of colony forming units (CFU)/mL and vegetative growth as mean value (±standard deviation) of colony diameters (Ø) of three independent experiments. All *D. viscosa* extracts were able to completely inhibit the germination of *M. canis* at concentration higher than 1 mg/mL. The germination of *A. fumigatus* was completely inhibited at concentrations higher than 10 mg/mL. *D. viscosa* extracts affect both *M. canis* vegetative growth and sporulation, being non-toxic for *M. canis* CD 1279 and *M. canis* CD 1447 only when ethanolic and 80% ethanolic of *D. viscosa* extracts were used at 1 mg/mL (Table 5). All *D. viscosa* extracts are toxic to *A. fumigatus,* except for the strains CD 1435 and CD 1441. In particular, all *D. viscosa* extracts at a concentration of 1 mg/mL are non-toxic for CD 1435, with the exception of 80% ethanolic extract which is not toxic for the *A. fumigatus* CD 1441 at this concentration.

The present study shows that all *D. viscosa* extracts significantly decrease the vegetative growth, germination, conidia production of both *M. canis* and *A. fumigatus,* thus confirming previous results against dermatophytes or other fungal species (i.e., *Cladosporium cucumerinum, Botrytis cinerea*, *Pseudoperonospora cubensis*, *Phytophthora infestans*, *Erysiphe graminis* and *Puccinia helianthi* [5,22]. However, all extracts evinced a concentration-dependent inhibitory activity, which varies accordingly to fungal genus. In fact, the *A. fumigatus* strains seems to be less susceptible than *M. canis* as previously reported using acetone extracts of *Arctotis arctotoides* [22]. Additionally the highest antifungal activity was observed with methanol extracts in both fungal species, thus suggesting the efficacy of both TPC and CQA content as antifungal drugs [21,27]. The mechanism of action of phenolic compounds against fungi was previously explained by several studies and might be due to the membrane lipid perturbation. Sung and Lee (2010) [28] demonstrated that phenolic acids might cause disruption of ion transport, whereas Teodoro et al. (2015) [20] indicated that the hydroxyl group and carboxylic acid groups of pheonlic compounds plays an important role in destabilizing the fungal cytoplasmic membrane. Even if the low toxicity values of *D. viscosa* methanolic extract in one strain of *A. fumigatus* need to be confirmed, the herein obtained results, suggested that concentrations higher than 1 mg/mL should be employed in controlling *A. fumigatus* strains. The antifungal activity against *Malassezia* yeasts, *M. canis* and *A. fumigatus* is of interest since the control of these infections is the subject of debate in the scientific community. In particular, *Malassezia* yeast infections in animals, mainly dogs, may be unresponsive to antifungal therapy and the animals usually have recurrences thus requiring multiple drug regimens [24]. The treatment of *M. canis* infections in animals is mandatory because of the zoophilic nature of this fungus, but it is not always possible in animals used for food production [28]. 

Finally, the high azoles resistance phenomena registered in *Aspergillus* spp. strains also suggests the usefulness of studies on new antifungal drugs [30]. All these findings promote the employment of drugs of plant origin.

## 3. Materials and Methods

### 3.1. Chemical and Reagents

Vanillin (C_3_H_8_O_3_), catechin (C_15_H_14_O_6_), Folin-Ciocalteu reagent, sodium carbonate (Na_2_CO_3_), gallic acid (C_7_H_6_O_5_), aluminum chloride (AlCl_3_), potassium acetate (C_2_H_3_KO_2_), rutin (C_27_H_30_O_16_), quercetin (C_15_H_10_O_7_), sodium molybdate dihydrate (Na_2_MoO_4_), dipotassium hydrogen phosphate (K_2_HPO_4_), potassium dihydrogen phosphate (KH_2_PO_4_), chlorogenic acid (C_16_H_18_O_9_) were purchased from Sigma**-**Aldrich**^®^** (Steinheim, Germany). Solvents of analytical and HPLC grade were purchased from Carlo Erba Reactif-CDS (Val de Reuil, France).

### 3.2. Plant Material

The leaves of the plants were collected in June 2015 from uncultivated land in Sidi Thabet, located in the North East of Tunisia (latitude 36°55′45″ N, longitude 10°06′02.10″ E, altitude 30 m).

### 3.3. Preparation of Extracts

Dried and ground leaves (10 g) were macerated in four different solvents (ethanol (80% and 100%), methanol and butanol) (10:100 *w*/*v*) for 48 h with shaking at room temperature. The extracts were filtered with Whatman No. 1 filter paper and the filtrate evaporated to dryness using a rotary evaporator. In order to test the antimicrobial activities, the samples were solubilized in dimethyl sulfoxide (DMSO) to obtain concentrations of 1, 5, 10 and 50 mg/mL.

### 3.4. Phytochemical Screening

#### 3.4.1. Condensed Tannins Content (CTC)

The CTC was determined as previously described [31]. In particular, 0.5 mL of extract was condensed using 3 mL of vanillin at 4% in methanol and 1.5 mL of concentrated hydrochloric acid (HCl). The mixture was kept in the dark for 15 min at 20 °C and the CTC were measured using a Jenway 6300 spectrophotometer (Cole-Parmer, Staffordshire, UK) at absorbance of 500 nm. The CTC was calculated from calibration curve using catechin (CAE) as a standard and results were expressed as milligrams of catechin equivalent per gramm (g) of dry extract (mg CAE/g).

#### 3.4.2. Total Phenol Content (TPC)

The TPC was determined using the Folin-Ciocalteu method [32]. Briefly, 0.5 mL of each dissolved extract was mixed with 2.5 mL of Folin-Ciocalteu reagent in each test tube. After 4 min, 2 mL of saturated sodium carbonate (Na_2_CO_3_) solution (7.5%) was added to the mixture. The reaction mixtures were incubated for 2 h. Methanol was used as the blank. All assays were conducted in triplicate and the results were averaged. The TPC was calculated from a calibration curve using gallic acid (GAE) as the standard and the results were expressed as milligrams of gallic acid equivalent per gram of extract (mg GAE/g).

#### 3.4.3. Total Flavonoid Content (TFC)

The TFC was quantified using the aluminum chloride colorimetric assay with slight modifications [33]. In brief, 0.5 mL of each solution extract was mixed with 1.5 mL methanol, 0.1 mL of 10% aluminum chloride, 0.1 mL of 1 mol/L potassium acetate solution and 2.8 mL distilled water. The mixture was allowed to stand for 15 min, and absorbance was measured at 415 nm. All assays were conducted in triplicate and the results were averaged. The TFC was calculated from a calibration curve using quercetin (QE) as the standard, and the result was expressed as mg of quercetin equivalent per gram dry extract (mg QE/g).

#### 3.4.4. Caffeoylquinic Acid (CQC) Content

The CQC content of extracts was quantified using the molybdate colorimetric method [34]. Sodium molybdate (16.5 g), dipotassium hydrogen (8.0 g) phosphate, and potassium dihydrogen phosphate (7.9 g) were dissolved in 1 liter of deionized water to prepare the molybdate reagent. For each *I. viscosa* extract solution 0.3 mL was mixed with 2.7 mL of molybdate reagent. The mixture was incubated at room temperature for 10 min. Absorbance was measured at 370 nm. All assays were conducted in triplicate and the results were averaged. The CQC was calculated from a calibration curve using chlorogenic acid (ChlA) as the standard and the result was expressed as mg of ChlA equivalent per g dry extract (mg ChlA/g).

### 3.5. Characterization of PhenolicCcompounds by HPLC-PDA-ESI-MS

The phenolic compounds present in methonolic extract were tentatively identified using the chromatographic separation method as previously reported [4].

Chromatographic separation was performed on an Alliance e2695 HPLC system (Waters, Bedford, MA, USA) equipped with a RP-xTerra MS column (150 × 4.6 mm i.d., 3.5 μm particle size), photodiode array detector (PDA) and interfaced with a triple quadruple mass spectrometer (MSD 3100, Waters) fitted with an ESI ion source. The sample (20 μL) was eluted through the column with a gradient mobile phase consisting of A (0.1% formic acid) and B (acetonitrile acidified with formic acid 0.1%) with a flow rate of 0.5 mL/min. The following multistep linear solvent gradient was used: 0–40 min: 14–26% B; 40–60 min: 15% B; 60–75: 0% B; 75–80 min: 14% B. The HPLC-PDA-ESI-MS chromatogram spectral data were stored and processed with Masslynx 4.1 data system. Each peak in the chromatogram was accomplished in a single chromatographic run in order to be identified [34].

### 3.6. Antibacterial and Antifungal Activities

#### 3.6.1. Bacterial Strains

Five reference bacterial strains, including Gram-positive (i.e., *Staphylococcus aureus* ATCC 6538, *Enterococcus feaciu*m ATCC 19434*, Streptococcus agalactiae* ATCC 12386), and Gram-negative (i.e., *Escherichia coli* ATCC 8739, 29212 and *Salmonella typhimurium* ATCC 14028), were used to assess the antibacterial properties of the extracts.

#### 3.6.2. Fungal Strains

##### *Candida* spp. Strains

Three references strains of *Candida* (i.e., *Candida krusei* ATCC 6258*, Candida parapsilosis* ATCC 22019, *Candida albicans* ATCC 10231), and four *Candida albicans* strains (i.e., CD 1358, CD1378, CD 1407, CD 1408) isolated from cloaca of laying hens, were used to evaluate the anti-*Candida* activity of *I. viscosa* extracts. All strains were obtained from the fungal collection of the Department of Veterinary Medicine at the University of Bari (Aldo Moro, Italy).

##### *Malassezia* spp. Strains

A total of six *Malassezia* spp. strains (three *Malassezia pachydermatis* and three *Malassezia furfur*) were tested. Two reference strains (i.e., *M. pachydermatis* CBS1879 and *M. fufur* CBS1978), two strains isolated from dogs with dermatitis and/or otitis (i.e., *M. pachydermatis* CD 112 and CD 90), two *M. furfur* strains from human skin (i.e., *M. furfur* CD 1029), and one from a human blood stream infection (i.e., *M. furfur* CD 1006) were tested.

##### *Aspergillus fumigatus*strains

Three *A. fumigatus* strains (CD 1435, CD 1438 and CD 1441) were tested. All strains were isolated from the respiratory tract of critically ill human patients. All strains were obtained from the fungal collection of the Department of Veterinary Medicine at the University of Bari.

##### *Microsporum canis* strains

Three *M. canis* strains (CD 1243, CD 1447, and CD 1279), isolated with skin lesions from human, cat, and dog were tested, respectively. All strains were stored in the fungal collection of the Department of Veterinary Medicine at the University of Bari.

### 3.7. Determination of Antibacterial, Anti-Candida and Antifungal Activity of I. viscosa Extract

The antibacterial, anti-*Candida* and anti-*Malassezia* activities were evaluated by the disk diffusion method [36], whereas the antifungal activity of *I. viscosa* extracts against *M. canis* and *A. fumigatus* was assessed by studying the toxicity effect of the extract on vegetative growth and sporulation as well their effect on fungal germination.

#### 3.7.1. Toxicity Assay

The antifungal activity of *D. viscosa* extracts against *M. canis* and *A. fumigatus* was assessed as previously reported [37]. In particular, the antifungal properties of extracts were assessed by applying the following mathematical model in order to evaluate the degree of toxicity:

*T* = 20[VG] + 80[SR]/100 (1)

where: *T* is the degree of toxicity useful for the classification of the product; VG is the percentage of vegetative growth with respect to the control; SR is the percentage of sporulation with respect to the control. The product was classified, based on the T value, as: very toxic (0 ≤ *T* ≤ 30); toxic (31 ≤ *T* ≤ 45) moderately toxic (46 ≤ *T* ≤ 60); non-toxic (i.e., compatible) (*T* > 60) [36].

The *A. fumigatus* and *M canis* strains were sub-cultured onto PDA and incubated at 25 °C for 10 days before testing. Vegetative growth (VG) was measured by placing a mycelial plug (i.e., 5 mm in diameter) onto the center of a 90 mm Petri dish containing potato dextrose agar (PDA), with and without extract or DMSO (solvent control), and measuring the diameter of the colonies after incubation at 25 °C for 10 days. Sporulation was evaluated by collecting the spores from surface of fungi grown on the PDA with and without the extracts after 10 incubation days at 25 °C. Spores and mycelia were collected by scraping the surface of the plate with 4 mL of 20% tween 80 solution. The solution was filtered through sterile gauze to remove mycelia, and then centrifuged (3000 *g* × 5 min), washed twice in 1 mL of phosphate-buffered saline solution (PBS), and re-suspended in 1 mL of PBS. Numbers of spores were determined by quantitative plate counts of (CFU)/mL on PDA after incubation at 25 °C for 4 days [37].

#### 3.7.2. Effect of *D. viscosa* Extract on Fungal Germination

The effect of *D. viscosa* extracts on *M. canis* and *A. fumigatu*s germination was also measured, culturing fungi in SDA medium after 14 days at 25 °C and collecting spores and mycelia as reported above. The solution obtained was diluted in PBS to obtain an inoculum concentration of 10^7^ conidia/mL which was evaluated by quantitative plate counts of CFU/mL in PDA. Finally, a total of 100 µL of the fungal spore suspensions were cultured in PDA with and without different extract concentrations. The number of germinated spores were determined by counts of CFU/mL on PDA [38]. All experiments were performed in duplicate and repeated three times on different days.

### 3.8. Statistical Analysis

The results of toxicity assay on vegetative growth, sporulation and fungal germination were expressed as mean values (±standard deviation (SD)) of the three independent experiments. Vegetative growth (VG) was expressed as mean value of colony diameters after incubation and the sporulation and germination as mean values of Log_10_ CFU/mL. Results were statistically analyzed using one way analysis of variance (ANOVA). Significant differences were set at *p* < 0.05.

## 4. Conclusions

The employment of these extracts might be useful to treat infections that are life threatening (i.e., *Malassezia*) or infections that require a mandatory treatment (i.e., *M. canis* or *A. fumigatus*), thus providing another commercial validation of this weed and working towards reducing the hazards associated with excessive use of chemical products.

## Figures and Tables

**Table 1:** Condensed tannins, total polyphenols, total flavonoids, and caffeoylquinic acid content of different *D. viscosa* leaf extracts.

Polyphenols and Flavonoids Content	Ethanolic	Ethanolic 80%	Butanolic	Methanolic
**CTC(mgCAE/g extract)**	14.29 ± 1.30 ^a^	7.05 ± 1.6 ^b^	16.86 ± 1.62 ^c^	27.15 ± 2.21 ^d^
**TPC (mgGAE/g extract)**	117.58 ± 1.29 ^a^	123.39 ± 1.22 ^b^	75.34 ± 1.30 ^c^	123.07 ± 1.69 ^b^
**TFC (mgQE/g extract)**	57.79 ± 1.76 ^a^	49.23 ± 1.039 ^b^	58.03 ± 1.85 ^a^	30.86 ± 50 ^c^
**CQC (mgCGAE/g extract)**	71.85 ± 0.35 ^a^	73.13 ± 1.06 ^a^	57.11 ± 0.98 ^b^	87.61 ± 1.06 ^c^

Values followed by the same letter along the row are not significantly different (*p* < 0.05).

**Table 2:** Retention time (RT), wavelengths of maximum absorption (λmax), mass spectral data, relative occurrence, and tentative identification of phenolic compounds in methanolic extract of *D. viscosa* leaves.

Compound	RT (min)	λmax	[M − H]^−^	Fragment Ions	Proposed Structure	Occurrence
**1**	4.856	292sh–322	377	341 (100), 179, 119	Caffeic acid hexoside	++
**2**	5.346	292sh–322	341	191 (100), 137, 128	3-Caffeoylquinic acid	++
**3**	11.876	292sh–322	353	191 (100), 161	Chlorogenic acid	+++
**4**	13.856	292sh–322	353	191, 161 (100)	4-Caffeoylquinic acid isomer	+
**5**	17.222	293–310	467	163 (100)	Coumaric acid derivative	+
**6**	22.365	-	429	267 (100), 173, 161	Feruloyl caffeolglycerol	+
**7**	27.982	360, 262	463	301 (100), 331, 255	Quercetin hexoside	+
**8**	28.007	293sh–321	463	301(100)	Hydroxyluteolin hexoside	+
**9**	30.452	293sh–354	477	301 (100)	Quercetin glucuronide	+
**10**	30.714	294sh–354	477	301 (100), 161	Quercetin glucuronide	+
**11**	32.888	292sh–322	353	191 (100), 179, 161	5-Caffeoylquinic acid	+
**12**	33.463	292sh–322	353	191 (100), 179 (32)	Dicaffeoylquinic acid isomer	++++
**13**	37.98	292–322	353	191 (78), 179 (100), 161 (80)	Caffeoylquinic acid isomer	+++
**14**	44.561	290, 320	339	135 (100)	Caffeoyl glucose	+++
**15**	47.234	253–349	329	314 (100), 299 (80), 285 (70), 271 (53), 243 (50)	Quercetin-dimethyl ether isomer	+++
**16**	47.395	253–349	329	314 (100), 299 (85) 271 (75), 241 (40)	Quercetin-dimethyl ether isomer	+++
**17**	49.315	253–349	329	314 (100), 299 (85), 285, 243	Quercetin-dimethyl ether isomer	+
**18**	55.566	278	493	289 (40), 165 (100), 139 (80)	Catechin glucoside	+++

+: low in abundance; ++: moderate in abundance; +++: high in abundance); ++++: very high in abundance.

**Table 3:** Antibacterial properties of extracts under study, expressed as diameter of inhibition halo (in mm) versus several strains.

Bacterial spps.	Concentration (mg/mL)	Ethanol	Ethanol 80%	Butanol	Methanol
*Eshershia coli*	50	12±1.41 ^a^	11.5 ± 0.70 ^a^	12.5 ± 0.70 ^a^	12 ± 0.70 ^a^
	10	11 ± 1.41 ^a^	10 ± 0.0 ^a^	10.5 ± 0.0 ^a^	10 ± 0.0 ^a^
*Sal Salmonella typhimurium*	50	10.5 ± 0.70 ^a^	9.5 ± 0.70 ^a^	10.5 ± 0.70 ^a^	10 ± 0.0 ^a^
	10	9.5 ± 0.70 ^a^	0 ± 0.0 ^b^	9.5 ± 0.70 ^a^	9.5 ± 0.0 ^a^
*Enterococcus feacium*	50	34 ± 1.41 ^a^	28.5 ± 0.0 ^b^	34.5 ± 0.70 ^a^	34.5 ± 0.7 ^a^
	10	30 ± 0.0 ^a^	25 ± 0.0 ^b^	28 ± 0.0 ^c^	29 ± 0.0 ^d^
*Streptococcus agalactiae*	50	28 ± 1.41 ^a^	28 ± 1.41 ^a^	29 ± 1.41 ^a^	29 ± 1.41 ^a^
	10	18.5 ± 0.70 ^a^	17 ± 0.0 ^a^	21.5 ± 1.41 ^b^	18 ± 1.14 ^a^
*Staphylococus aureus*	50	25 ± 0.0 ^a^	25 ± 0.0 ^a^	22.5 ± 0.70 ^b^	20 ± 0.0 ^c^
	10	13.5 ± 0.70 ^a^	10 ± 0.0 ^b^	13 ± 1.41 ^a^	11 ± 0.0 ^c^

Values followed by the same superscript along the row are not significantly different (*p* < 0.05).

**Table 4:** *Anti-Candida* and *Anti-Malassezia* properties of extracts under study, expressed as diameter of inhibition halo (in mm) versus several strains.

Candida and *Malassezia* spp.	Concentration (mg/mL)	Ethanol	Ethanol 80%	Butanol	Methanol
***Candida prapsilosis* ATCC 22019**	50	10.25 ± 0.58 ^a^	9.66 ± 1.52 ^a^	8.75 ± 1.73 ^a^	10.75 ± 0.95 ^a^
	10	8.66 ± 1.73 ^a^	8.5 ± 1.73 ^a^	8.66 ± 1.73 ^a^	10 ± 0.95 ^a^
***Candida krusei* ATCC 6258**	50	10 ± 1.41 ^a^	10.5 ± 0.57 ^a^	10 ± 1 ^a^	10 ± 0.0 ^a^
	10	9.5 ± 0.7 ^a^	10 ± 0.0 ^a^	9 ± 0.82 ^a^	10 ± 0.0 ^a^
***Candida albicans* ATCC 10231**	50	13.5 ± 0. 70 ^a^	13.5 ± 0.70 ^a^	14.5 ± 0.70 ^a^	14.5 ± 0.70 ^a^
	10	12 ± 0.0 ^a^	11.5 ± 0.70 ^a^	13 ± 0.00 ^a^	12 ± 1.41 ^a^
***Candida albicans* CD 1358**	50	10.5 ± 0.57 ^a^	11 ± 0.00 ^a^	10.25 ± 0.5 ^a^	10 ± 2.0 ^a^
	10	10.25 ± 0.5 ^a^	10 ± 0.57 ^a^	9.5 ± 0.57 ^a^	9.5 ± 2.0 ^a^
***Candida albicans* CD 1378**	50	10.25 ± 0.5 ^a^	11.0 ± 0 ^b^	10 ± 0.0 ^a^	10 ± 0.81 ^a^
	10	10 ± 0.5 ^a^	10.33 ± 0.0 ^a^	10 ± 0.5 ^a^	10 ± 0.0 ^a^
***Candida albicans* CD 1407**	50	10.66 ± 0 ^a^	10.33 ± 1.89 ^a^	10.75 ± 0.5 ^a^	11 ± 0.81 ^a^
	10	10 ± 0.5 ^a^	8.25 ± 1.89 ^a^	10.33 ± 0.57 ^a^	9.66 ± 0.57 ^a^
***Candida albicans* C13F3A**	50	9.5 ± 1.91 ^a^	10.5 ± 0.57 ^a^	10.75 ± 0.5 ^a^	11 ± 0.81 ^a^
	10	7 ± 1.15 ^a^	10 ± 0.57 ^a^	6.66 ± 0.57 ^a^	9.66 ± 0.57 ^a^
***Malassezia pachydermatis* CBS1879**	50	10 ± 0.0 ^a^	10 ± 0.0 ^a^	10.33 ± 0.57 ^a^	11 ± 00 ^a^
	10	9.33 ± 1.15 ^a^	9.33 ± 0.57 ^a^	9.66 ± 1.52 ^a^	9.33 ± 0.57 ^a^
***Malassezia pachydermatis* CD 112**	50	10.33 ± 0.57 ^a^	10.66 ± 0.57 ^a^	9.33 ± 1.15 ^a^	10.66 ± 0.57 ^a^
	10	7.66 ± 0.57 ^a^	7.66 ± 0.57 ^a^	7 ± 0.0 ^a^	10.33 ± 0.57 ^b^
***Malassezia pachydermatis* CD 90**	50	10.33 ± 1.55 ^a^	10.33 ± 0.57 ^a^	9.66 ± 0.57 ^a^	9.66 ± 0.57 ^a^
	10	0 ± 0.0 ^a^	7.66 ± 0.57 ^b^	7.33 ± 0.57 ^b^	9.33 ± 0.57 ^c^
***Malassezia furfur* CBS1978**	50	10.66 ± 1.54 ^a^	10.33 ± 1.52 ^a^	8.33 ± 0.57 ^a^	9.66 ± 1.15 ^a^
	10	0 ± 0.0 ^a^	7 ± 0.0 ^b^	0 ± 0.0 ^a^	8 ± 1.0 ^b^
***Malassezia furfur* CD 1006**	50	9.33 ± 0.57 ^a^	9.66 ± 1.52 ^a^	8.33 ± 1.52 ^a^	9 ± 1.73 ^a^
	10	0 ± 0.0 ^a^	0 ± 0.0 ^a^	0 ± 0.0 ^a^	8 ± 1.0 ^b^
***Malassezia furfur *CD 1029**	50	8 ± 1.0 ^a^	9 ± 0.0 ^a^	0 ± 0.0 ^b^	9 ± 1.0 ^a^
	10	0 ± 0.0	0 ± 0.0	0 ± 0.0	0 ± 0.0

Values followed by the same superscript letter along the row are not significantly different (*p* < 0.05).

**Table 5:** Effects of *D. viscosa* extracts on conidia germination, vegetative growth and sporulation of *M. canis*. Degree of toxicity (*T* value) was also reported.

	[C] mg/mL	Ethanol				Ethanol 80%				Butanol				Methanol			
Strains		Germination (Log_10_ CFU/mL)	Growth (Ø cm)	Sporulation (Log_10_ CFU/mL)	*T* Value	Germination (Log_10_ CFU/mL)	Growth (Ø cm)	Sporulation (Log_10_ CFU/mL)	*T* Value	Germination (Log_10_ CFU/mL)	Growth (Ø cm)	Sporulation (Log_10_ CFU/mL)	*T* Value	Germination (Log_10_ CFU/mL)	Growth (Ø cm)	Sporulation (Log_10_ CFU/mL)	*T* Value
**CD 1279**	50	0	0	0	**0**	0	0	0	**0**	0	0	0	**0**	0	0	0	**0**
	10	0	0	0	**0**	0	0	0	**0**	0	0	0	**0**	0	0	0	**0**
	5	0	2.4 ± 0.3	3.54 ± 0.1	**14.71**	0	2.55 ± 0.1	4.06 ± 0.1	**23.08**	0	2.5 ± 0.1	3.47 ± 0.1	**14.54**	0	1.8 ± 0.1	2.97 ± 0.1	**9.31**
	1	3.72 ± 0.1	3.65 ± 0.4	4.79 ± 0.1	**77.78**	3.87 ± 0.1	3.85 ± 0.1	4.77 ± 0.1	**76.91**	3.56 ± 0.2	3.5 ± 0.1	4.58 ± 0.1	**53.55**	3.49 ± 0.1	2.55 ± 0.1	4.43 ± 0.1	**38.45**
	**Control**	4.30 ± 0.1	5.45 ± 0.1	4.91 ± 0.1	**100**	4.30 ± 0.2	5.45 ± 0.1	4.91 ± 0.1	**100**	4.30 ± 0.2	5.45 ± 0.1	4.91 ± 0.1	**100**	4.3 ± 0.2	5.45 ± 0.1	4.91 ± 0.1	**100**
**CD 1243**	50	0	0	0	**0**	0	0	0	**0**	0	0	0	**0**	0	0	0	**0**
	10	0	0	0	**0**	0	0	0	**0**	0	0	0	**0**	0	0	0	**0**
	5	0	2.1 ± 0.14	3.13 ± 0.1	**10.62**	0	2.25 ± 0.1	3.41 ± 0.04	**13.85**	0	1.85 ± 0.1	3.06 ± 0.1	**9.52**	0	1.85 ± 0.1	2.86 ± 0.1	**8.61**
	1	3.73 ± 0.1	2.55 ± 0.1	4.22 ± 0.2	**48.97**	3.81 ± 0.1	2.85 ± 0.1	4.22 ± 0.02	**45.41**	3.24 ± 0.1	2.85 ± 0.1	3.79 ± 0.1	**25.71**	3.24 ± 0.1	2.35 ± 0.1	3.43 ± 0.1	**15.11**
	**C**	4.18 ± 0.1	5.55 ± 0.1	4.51±0.1	**100**	4.18 ± 0.1	5.55 ± 0.1	4.51±0.1	**100**	4.18 ± 0.1	5.55 ± 0.1	4.51 ± 0.1	**100**	4.18 ± 0.1	5.55 ± 0.1	4.51 ± 0.1	**100**
**CD 1447**	50	0	0	0	0	0	0	0	0	0	0	0	0	0	0	0	0
	10	0	0	0	0	0	0	0	0	0	0	0	0	0	0	0	0
	5	0	2.5 ± 0.1	3.36 ± 0.2	16.58	0	2.85 ± 0.1	3.55 ± 0.2	21.12	0	2.45 ± 0.1	3.81 ± 0.1	14.77	0	1.65 ± 0.1	2.81 ± 0.1	9.16
	1	3.85 ± 0.1	3.45 ± 0.1	4.43 ± 0.1	66.31	3.88 ± 0.3	3.8 ± 0.1	4.26 ± 0.1	17.19	2.52 ± 0.1	3 ± 0.1	4.29 ± 0.1	50.23	2.29 ± 0. 3	2.65 ± 0.1	4.26 ± 0.1	46.31
	C	4.46 ± 0.1	4.15 ± 1	4.61 ± 0.1	100	4.46 ± 0.1	4.15 ± 0.1	4.61 ± 0.2	100	4.46 ± 0.1	4.15 ± 0.1	4.61 ± 0.1	100	4.46 ± 0.1	4.15 ± 0.1	4.61 ± 0.1	100

* *T* value = very toxic (0 ≤ *T* ≤ 30); toxic (31 ≤ *T* ≤ 45); moderately toxic (46 ≤ *T* ≤ 60); non-toxic (*T* > 60); [C]: Concentration (mg/mL); C: ControlConcentration (mg/mL); C: Control.

**Table 6:** Effects of *D. viscosa* extracts on conidia germination, vegetative growth and sporulation of *A.fumigatus*. Degree of toxicity (*T* value) was also reported.

	[C] mg/mL	Ethanol				Ethanol 80%				Butanol				Methanol			
Strains		Germination (Log_10_ CFU/mL)	Growth (Ø cm)	Sporulation (Log_10_ CFU/mL)	*T* Value	Germination (Log_10_ CFU/mL)	Growth (Ø cm)	Sporulation (Log_10_ CFU/mL)	*T* Value	Germination (Log_10_ CFU/mL)	Growth (Ø cm)	Sporulation (Log_10_ CFU/mL)	*T* Value	Germination (Log_10_ CFU/mL)	Growth (Ø cm)	Sporulation (Log_10_ CFU/mL)	*T* Value
**CD 1435**	50	0	1.25 ±0.1	5.09 ± 0.2	**2.88**	0	1.55 ± 0.1	6.25 ± 0.2	**4.71**	0	1.35 ± 0.1	5.12 ± 0.2	**3.11**	0	1.15 ± 0.1	4.08 ± 0.3	**2.59**
	10	3.66 ± 0.1	2.35 ± 0.1	6.45 ± 0.2	**7.13**	3.51 ± 0.1	2.85 ± 0.1	6.81 ± 0.1	**13.62**	3.47 ± 0.3	3.35 ± 0.2	6.18 ± 0.1	**8.56**	3.46 ± 0.3	1.95 ± 0.1	5.08 ± 0.6	**4.75**
	5	4.06 ± 0.1	5.9 ± 0.1	6.72± 0.2	**19.33**	4.20 ± 0.1	6.25 ± 0.1	7.23 ± 0.0	**29.86**	4.03 ± 0.3	5.7 ± 0.14	6.51 ± 0.1	**16.44**	4.14 3 ± 0.1	5.35 ± 0.1	6.18±0.3	**14.45**
	1	5.21 ± 0.2	7.85 ± 0.1	7.75 ± 0.1	**73.53**	5.03 ± 0.1	8.15 ± 0.1	7.79 ± 0.1	**78.41**	4.97 ± 0.1	8.15 ± 0.1	7.74 ± 0.1	**73.71**	4. 7 ± 0.1	8.25 ± 0.1	7.66±0.1	**64.9**
	C	5.24 ± 0.1	8.9 ± 1.0	7.91 ± 0.1	**100**	5.24 ± 0.1	8.9 ± 1.0	7.91 ± 0.6	**100**	5.24 ± 0.1	8.95 ± 1.0	7.91 ± 0.1	**100**	5.24 ± 0.1	8.95 ± 1.1	7.91±0.1	**100**
**CD 1441**	50	0	1.2 ± 0.0	5.35 ± 0.2	**2.86**	0	1.65 ± 0.1	5.23 ± 0.2	**3.85**	0	1.2 ± 0	5.13 ± 0.3	**2.81**	0	1.1 ± 0	4. 67 ± 0.1	**2.54**
	10	3.55 ± 0.1	3.4 ± 0.3	6.59 ± 0.1	**9.93**	3.68 ± 0.1	3.4 ± 0.0	6.56 ± 0.1	**9.8**	3.19 ± 0.1	3.3 ± 0.5	6.17 ± 0. 2	**8.33**	2.67 ± 0.1	2.95 ± 0.1	5.64 ± 0.1	**8.25**
	5	4.07 ± 0.2	5.25 ± 0.1	6.96 ± 0.1	**17.2**	4.34 ± 0.1	5.4 ± 0.3	7.04 ± 0.10	**18.78**	3.95 ± 0.1	4.55 ± 0.2	6.84 ± 0.1	**14.34**	3.8 ± 0.1	4.4 ± 0.2	6.72 ± 0.1	**13.1**
	1	4.99 ± 0.1	7.55 ± 0.2	7.73 ± 0.5	**47.5**	5.06 ± 0.1	7.95 ± 0.1	7.9 ± 0.1	**63.81**	4.83 ± 0.1	7.4 ± 0.1	7.57 ± 0.1	**38.74**	4.66 ± 0.1	7.8 ± 0.1	7.47 ± 0.1	**34.82**
	**C**	5.35 ± 0.1	8.8 ± 0.1	8.15 ± 0.1	**100**	5.53 ± 0.1	8.8 ± 0.1	8.15 ± 0.1	**100**	5.35 ± 0.1	8.84 ± 0.1	8.15 ± 0.1	**100**	5.35 ± 0.1	8.8 ± 0.1	8.15 ± 0.1	**100**
**CD 1438**	50	0	1.25 ± 0.1	5.06 ± 0.1	**2.84**	0	1.6 ± 0	5.19 ± 1.1	**3.83**	0	0	0	**0**	0	1.2 ± 0.0	0.00	**0**
	10	4.3 ± 0.1	3.05 ± 0.2	5.76 ± 0.5	**7.2**	4.58 ± 0.1	3.8 ± 0.1	6.5 ± 0.2	**9.72**	2.67 ± 0.1	2.95 ± 0.1	5.85 ± 0.1	**6.94**	4.3 ± 0.1	2.55 ± 0.2	5.61 ± 0.1	**5.97**
	5	5.08 ± 0.1	5.55 ± 0.2	6.69 ± 0.1	**14.37**	5.19 ± 0.1	6.6 ± 0.1	7.78 ± 0.1	**23.54**	3.96 ± 0.1	3.95 ± 0.1	6. 84 ± 0.1	**12.23**	4.68 ± 0.1	3.6 ± 0.1	6.18 ± 0.2	**8.84**
	1	5.45 ± 0.1	7.65 ± 0.2	7.89 ± 0.1	**49.38**	5.76 ± 0.1	8.0 ± 0.3	7.78 ± 0.01	**41.49**	4.12 ± 0.1	7.62± 0.1	7.78 ± 0.1	**40.84**	5.3 ± 0	7.55 ± 0.2	7.69 ± 0.1	**40.73**
	C	**5.92 ± 0.1**	**8.95 ± 1.0**	**8.31 ± 0.2**	**100**	**5.92 ± 0.1**	**8.95 ± 1.0**	**8.31 ± 0.2**	**100**	**5.92 ± 0.1**	**8.95 ± 1.0**	**8.31 ± 0.2**	**100**	**5.92 ± 0.1**	**8.95 ± 1.0**	**8.31 ± 0.2**	**100**

## References

[1] Rossolini G.M., Arena F., Pecile P., Pollini S. (2014). Update on the antibiotic resistance crisis. Curr. Opin. Pharmacol..

[2] Çelik T.A., Aslantürk Ö.S. (2010). Evaluation of cytotoxicity and genotoxicity of *Inula viscosa* leaf extracts with *Allium* test. J. Biomed. Biotechnol..

[3] Parolin P., Scotta M.I., Bresch I. (2013). Biology of *Dittrichia viscosa*, a Mediterranean ruderal plant. Фyton..

[4] Mahmoudi H., Hosni K., Zaouali W., Amri I., Zargouni H., Hamida N.B., Kaddour R., Hamrouni L., Nasri M.B., Ouerghi Z. (2015). Comprehensive Phytochemical Analysis, Antioxidant and Antifungal Activities of *Inula viscosa* Aiton Leaves. J. Food Saf..

[5] Omezzine F., Daami-Remadi M., Rinez A., Ladhari A., Haouala R. (2011). In vitro assessment of *Inula* spp. organic extracts for their antifungal activity against some pathogenic and antagonistic fungi. Afr. J. Microbiol. Res..

[6] Assaf A.M., Amro B.I., Mashallah S., Haddadin R.N. (2016). Antimicrobial and anti-inflammatory potential therapy for opportunistic microorganisms. J. Infect. Dev. Ctries.

[7] Duek L., Kaufman G., Ulman Y., Berdicevsky I. (2004). The pathogenesis of dermatophyte infections in human skin sections. J. Infect..

[8] Bender J.B., Shulman S.A. (2004). Reports of zoonotic disease outbreaks associated with animal exhibits and availability of recommendations for preventing zoonotic disease transmission from animals to people in such settings. J. Am. Vet. Med. Assoc..

[9] Raad I.I., Graybill J.R., Bustamante A.B., Cornely O.A., Gaona-Flores V., Afif C., Graham D.R., Greenberg R.N., Hadley S., Langston A. (2006). Safety of long-term oral posaconazole use in the treatment of refractory invasive fungal infections. Clin. Infect. Dis..

[10] Cafarchia C., Figueredo L.A., Otranto D. (2013). Fungal diseases of horses. Vet. Microbiol..

[11] Cafarchia C., Figueredo L.A., Iatta R., Montagna M.T., Otranto D. (2012). In vitro antifungal susceptibility of *Malassezia pachydermatis* from dogs with and without skin lesions. Vet. Microbiol..

[12] Cafarchia C., Figueredo L.A., Iatta R., Colao V., Montagna M.T., Otranto D. (2012). In vitro evaluation of *Malassezia pachydermatis* susceptibility to azole compounds using E-test and CLSI microdilution methods. Med. Mycol..

[13] Seyedmousavi S., Guillot J., Arn P., De Hoog G.S., Mouton J.W., Melchers W.J.G., Verweij P.E. (2015). *Aspergillus* and aspergilloses in wild and domestic animals: A global health concern with parallels to human disease. Med. Mycol..

[14] Sefi M., Fetoui H., Makni M., Zeghal N. (2010). Mitigating effects of antioxidant properties of *Artemisia campestris* leaf extract on hyperlipidemia, advanced glycation end products and oxidative stress in alloxan-induced diabetic rats. Food Chem. Toxicol..

[15] Trimech I., Weiss E.K., Chedea V.S., Marin D., Detsi A., Ioannou E., Roussis V., Kefalas P. (2014). Evaluation of anti-oxidant and acetylcholinesterase activity and identification of polyphenolics of the invasive weed *Dittrichia viscosa*. Phytochem. Anal..

[16] Negi P.S., Jayaprakasha G. (2003). Antioxidant and Antibacterial Activities of *Punica granatum* Peel Extracts. J. Food Sci..

[17] Geng H.-M., Zhang D.-Q., Zha J.-P., Qi J.-L. (2007). Simultaneous HPLC Determination of Five Flavonoids in Flos Inulae. Chromatographia.

[18] Naeem I., Saddiqe Z., Patel A., Hellio C. (2010). Analysis of flavonoid and antimicrobial activity of extracts of *Hypericum perforatum*. Asian J. Chem..

[19] Danino O., Gottlieb H.E., Grossman S., Bergman M. (2009). Antioxidant activity of 1,3-dicaffeoylquinic acid isolated from *Inula viscosa*. Food Res. Int..

[20] Teodoro G.R., Ellepola K., Seneviratne C.J., Koga-Ito C.Y. (2015). Potential use of phenolic acids as anti-*Candida* agents: A review. Front. Microbiol..

[21] Cushnie T.P.T., Lamb A.J. (2005). Antimicrobial activity of flavonoids. Int. J. Antimicrob. Agents.

[22] Otang W.M., Grierson D.S., Ndip R.N. (2011). The effect of the acetone extract of *Arctotis arctotoides* (Asteraceae) on the growth and ultrastructure of some opportunistic fungi associated with HIV/AIDS. Int. J. Mol. Sci..

[23] Silhavy T.J., Kahne D., Walker S. (2010). The bacterial cell envelope. Cold Spring Harb. Perspect. Biol..

[24] Velegraki A., Cafarchia C., Gaitanis G., Iatta R., Boekhout T. (2015). *Malassezia.* Infections in Humans and Animals: Pathophysiology, Detection, and Treatment. PLoS Pathog..

[25] Berenji F., Rakhshandeh H., Ebrahimipour H. (2010). In vitro study of the effects of henna extracts (*Lawsonia inermis*) on Malassezia species. Jundishapur J. Microbiol..

[26] Filip R., Davicino R.,  Anesini C. (2010). Antifungal activity of the aqueous extract of Ilex paraguariensis against malassezia furfur. Phyther. Res..

[27] Von Nussbaum F., Brands M., Hinzen B., Weigand S., Häbich D. (2006). Antibacterial natural products in medicinal chemistry–Exodus or revival?. Angew. Chem. Int. Ed..

[28] Sung W.S., Lee D.G. (2010). Antifungal action of chlorogenic acid against pathogenic fungi, mediated by membrane disruption. Pure Appl. Chem..

[29] Magwedere K., Hemberger M.Y., Hoffman L.C., Dziva F. (2012). Zoonoses: A potential obstacle to the growing wildlife industry of Namibia. Infect. Ecol. Epidemiol..

[30] Tanwar J., Das S., Fatima Z., Hameed S. (2014). Multidrug Resistance: An Emerging Crisis. Interdiscip. Perspect. Infect. Dis..

[31] Chavan U., Shahidi F., Naczk M. (2001). Extraction of condensed tannins from beach pea (*Lathyrus maritimus* L.) as affected by different solvents. Food Chem..

[32] Meda A., Lamien C.E., Romito M., Millogo J., Nacoulma O.G. (2005). Determination of the total phenolic, flavonoid and proline contents in Burkina Fasan honey, as well as their radical scavenging activity. Food Chem..

[33] Zheng C.J., Yoo J.-S., Lee T.-G., Cho H.-Y., Kim Y.-H., Kim W.-G. (2005). Fatty acid synthesis is a target for antibacterial activity of unsaturated fatty acids. FEBS Lett..

[34] Chan E.W.C., Lim Y.Y., Ling S.K., Tan S.P., Lim K.K., Khoo M.G.H. (2009). Caffeoylquinic acids from leaves of *Etlingera* species (Zingiberaceae). LWT–Food Sci. Technol..

[35] Nyau V., Prakash S., Rodrigues J., Farrant J. (2015). HPLC-PDA-ESI-MS Identification of Polyphenolic Phytochemicals in Different Market Classes of Common Beans (*Phaseolus vulgaris* L.). Int. J. Biochem. Res. Rev..

[36] Dhouioui M., Boulila A., Jemli M., Schiets F., Casabianca H., Zina M.S. (2016). Fatty Acids Composition and Antibacterial Activity of *Aristolochia longa* L. and *Bryonia dioïca* Jacq. Growing Wild in Tunisia. J. Oleo Sci..

[37] Immediato D., Aguiar L., Iatta R., Camarda A., Lira R., De Luna N., Giangaspero A., Brandão-filho S.P., Otranto D., Cafarchia C. (2016). Veterinary Parasitology Essential oils and *Beauveria. bassiana* against *Dermanyssus gallinae* (Acari: Dermanyssidae): Towards new natural acaricides. Vet. Parasitol..

[38] Tamai M.A., Alves S.B., Lopes R.B., Faion M., Padulla L.F.L. (2002). Toxicidade De Produtos Fitossanitários para Beauveria Bassiana (Bals.) Vuill. Arq. Inst. Biol. (Sao Paulo).

